# More Than Meets the Eye: A Case of Endogenous Panophthalmitis

**DOI:** 10.7759/cureus.77137

**Published:** 2025-01-08

**Authors:** Bruna Cunha, Pedro Gil, Inês Ludovico, Catarina Mota, Lívio Costa

**Affiliations:** 1 Ophthalmology, Unidade Local de Saúde de São José, Lisbon, PRT

**Keywords:** bacterial endogenous endophthalmitis, colonic-type adenocarcinoma, negative blood culture, panophthalmitis, systemic antibiotics

## Abstract

Panophthalmitis is a severe condition involving not only the eye but also the surrounding orbital soft tissues. An endogenous source of panophthalmitis is rare and requires a thorough investigation to identify the infection's origin, which can be challenging. We present a rare case of a 65-year-old male patient with no systemic symptoms who presented to the emergency room with left-eye panophthalmitis, ultimately revealing occult colorectal cancer. Although vision could not be preserved, as is often the case in such severe presentations, the tumour was surgically removed, significantly improving the patient's life prognosis. This case highlights the importance of a multidisciplinary approach in managing complex patients.

## Introduction

Endophthalmitis is an ophthalmic emergency with potentially devastating visual and even life-threatening consequences. It is defined as an intraocular infection involving the internal structures of the eye, associated with progressive vitreous inflammation that affects both the anterior and posterior eye chambers [1]. When orbital cellulitis is also present, characterized by a rapidly progressing suppurative process, the condition is referred to as panophthalmitis [2]. Most cases arise from external sources, such as previous surgery, trauma, or an infected cornea, and are classified as exogenous endo/panophthalmitis [3].

An endogenous source of endo/panophthalmitis, which accounts for only 2%-8% of all cases [4-6], results from hematogenous spread of microorganisms due to a breakdown in the blood-ocular barrier [7]. This form is often associated with systemic risk factors such as recent hospitalization, diabetes mellitus, immunosuppression (e.g., malignancy, neutropenia, or HIV infection), intravenous drug use, or indwelling catheters. The prognosis in such cases is frequently poor [7-9].

Endogenous panophthalmitis may, at times, serve as the first manifestation of an undiagnosed systemic disease, even in the absence of systemic symptoms or positive cultures for infective pathogens. In this report, we present a rare case of endogenous panophthalmitis secondary to an undiagnosed rectal neoplasm with a rectovesical fistula in a patient with no systemic symptoms at presentation. This case underscores the importance of an exhaustive diagnostic workup, which can be crucial for both visual and life prognosis.

## Case presentation

A 65-year-old male patient was transferred to our emergency department with a five-day history of left ocular pain, red eye, decreased visual acuity, and periorbital oedema. He denied trauma, prior ophthalmological history, recent ocular surgery, or animal contact. Systemic symptoms, including fever, night sweats, weight loss, and arthralgias, were absent. His medical history included gastritis with a peptic ulcer, managed with a proton pump inhibitor. He was a smoker and reported occasional alcohol consumption.

**Figure 1:**
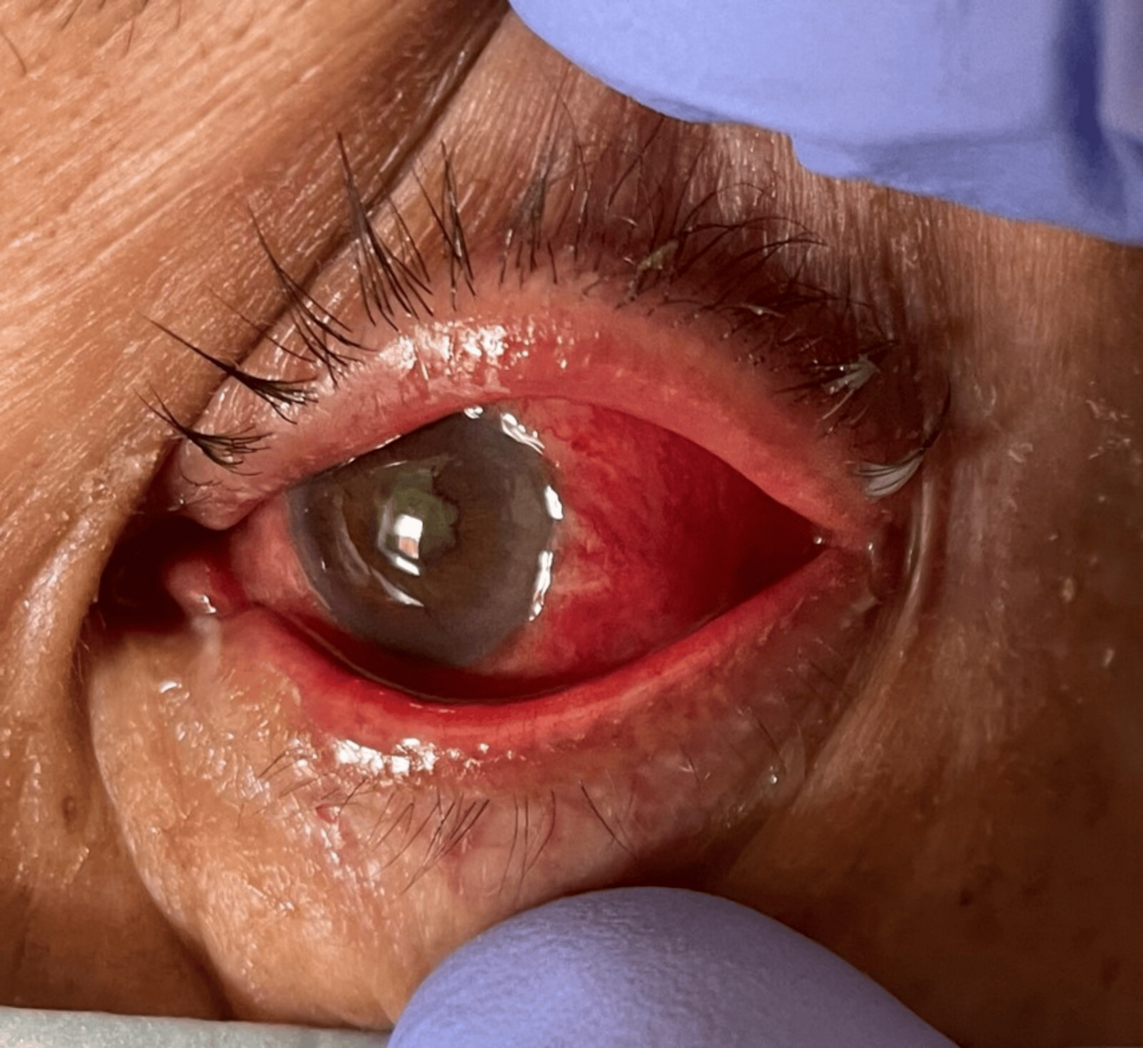
On examination, the left eye revealed proptosis, periorbital oedema, and complete ophthalmoplegia.

On examination, the best-corrected visual acuity was 20/20 in the right eye (OD) and no light perception in the left eye (OS), with no pupillary reflex, complete ophthalmoplegia, proptosis, and periorbital oedema (Figure 1).

**Figure 2:**
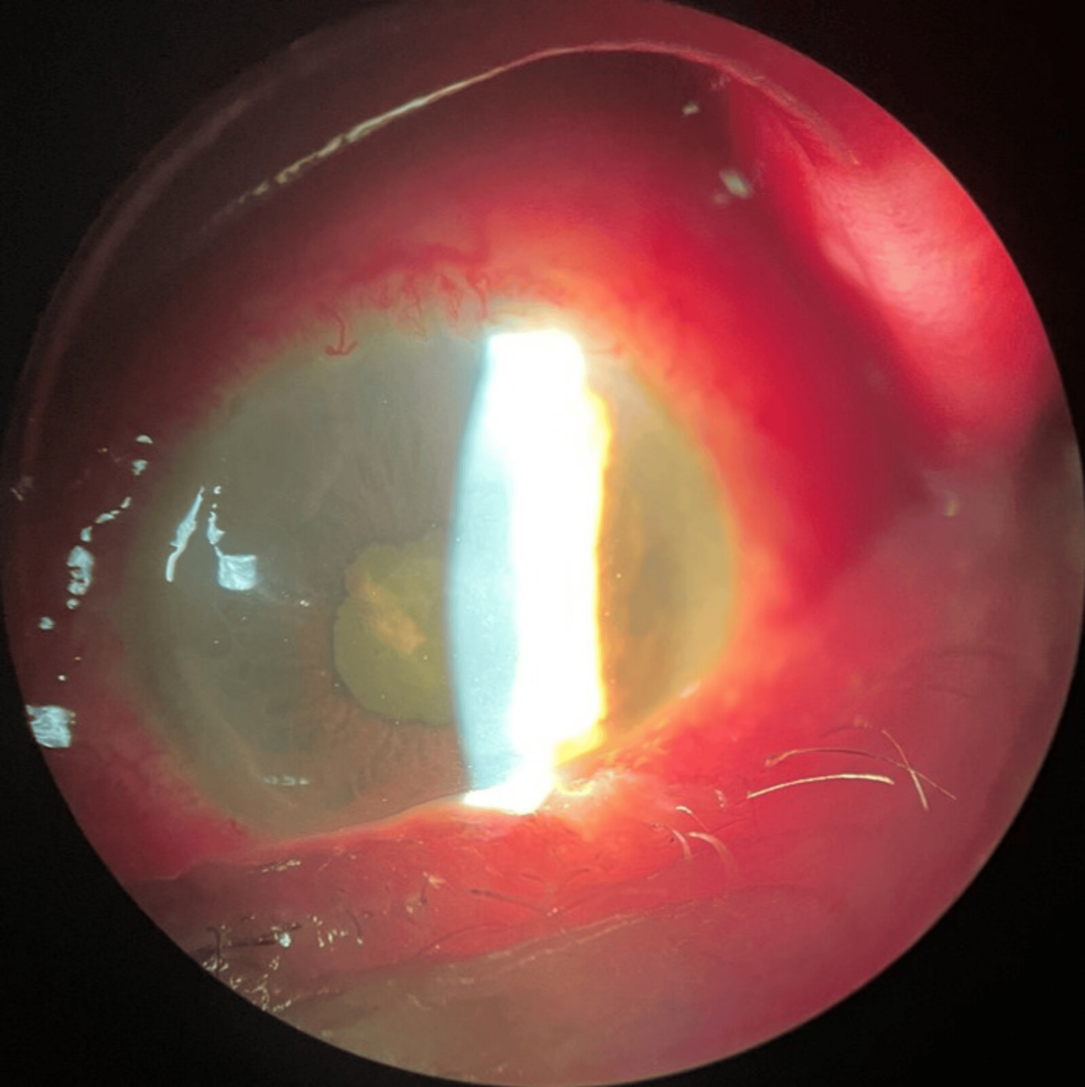
Biomicroscopy revealed chemosis, vascular injection, corneal oedema, anterior chamber fibrin reaction, and posterior synechiae.

Intraocular pressure was rock-hard on digital palpation. Biomicroscopy revealed chemosis, vascular injection, corneal oedema, anterior chamber fibrin reaction, and posterior synechiae (Figure 2). 

**Figure 3:**
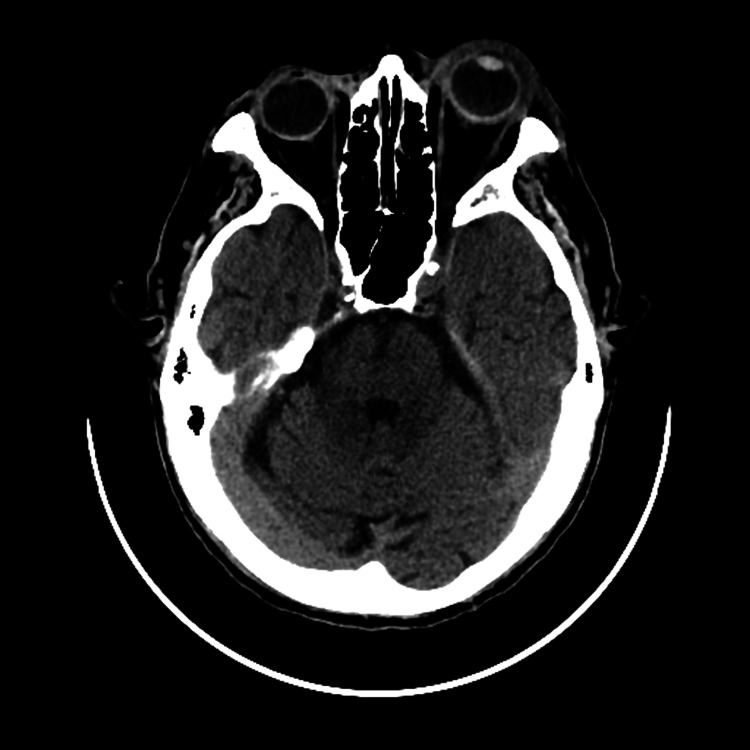
Head and orbital CT scan revealed, on the left side, orbital cellulitis, including a spontaneous hyperdense area interior to the orbital septum with contrast enhancement, retrobulbar fat densification, proptosis with optic nerve stretching, and chorioretinal thickening.

Fundus examination revealed dense vitritis with dispersed vitreous opacities, precluding retina visualization. B-scan ultrasonography demonstrated diffuse vitreous opacities and chorioretinal and scleral thickening. A head and orbital CT scan (Figure 3) revealed findings consistent with orbital cellulitis, including a spontaneous hyperdense area interior to the orbital septum with contrast enhancement, retrobulbar fat densification, proptosis with optic nerve stretching, and chorioretinal thickening. No cavernous sinus involvement or other significant abnormalities were identified. Computed tomography angiography and venography were unremarkable.

Laboratory tests revealed neutrophilic leukocytosis, hyponatremia, elevated C-reactive protein, and erythrocyte sedimentation rate (Table 1).

**Table 1:** Summary of blood tests performed

Test	Results	Reference values
Haemoglobin, x10g/L	12.8	13.0-17.0
Leukocytes, x10^9/L	13.42	4.5-11.0
Neutrophiles, x10^9/L	11.19	2.0-8.5
Platelets, x10^9/L	310	150-450
Prothrombin time, seconds	15.4	9.4-12.5
Activated partial thromboplastin time, seconds	35.6	25.1-36.5
Glucose, mg/dL	98	60-100
Urea, mg/dL	29	18.0-55.0
Creatinine, mg/dL	0.76	0.72-1.25
Sodium, mEq/L	132	136-145
Potassium, mEq/L	3.9	3.5-5.1
Chloride, mEq/L	94	98-107
C-reactive protein, mg/L	217	<5.0
Erythrocyte sedimentation rate, mm/h	62	<11
Anti-nuclear antibodies	Negative	-
Anti-dsDNA antibodies	Negative	-
Anti-neutrophil cytoplasm antibodies	Negative	-
HIV 1+2 antibodies	Negative	-
Venereal disease research laboratory (VDRL) test	Negative	-
*Treponema pallidum* hemagglutination assay (TPHA)	Negative	-
Hepatitis B surface (HBs) antigen	Negative	-
Hepatitis B core (HBc) antibodies	Negative	-
Hepatitis C (HC) total antibodies	Negative	-
Anti-*Toxoplasma gondii* antibodies (IgG)	Negative	-
Anti-*Toxoplasma gondii* antibodies (IgM)	Negative	-
Interferon-gamma release assay	Negative	-

Tympanic temperature was 37.2°C. Based on the clinical suspicion of endogenous panophthalmitis of unknown origin, intravenous vancomycin (1 g twice a day (BID)) and ceftriaxone (1 g BID) were initiated, with oral prednisolone (40 mg BID) added 24 hours later.

**Figure 4:**
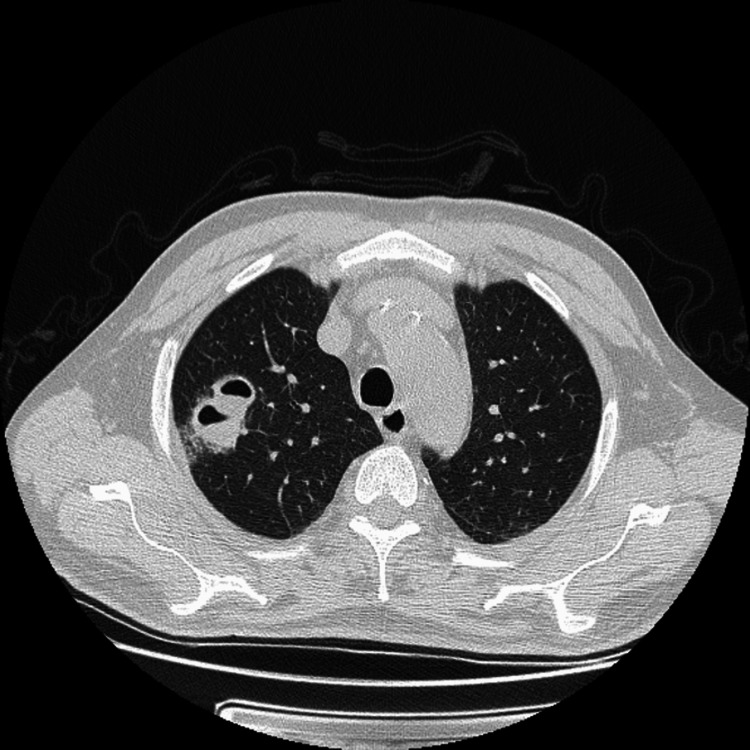
A thoracic CT scan revealed bilateral pulmonary cavitations at the superior lobes

During hospitalization, extensive laboratory investigations were conducted, with negative infectious and autoimmune serologies (Table 1). Transthoracic echocardiography revealed no signs of endocarditis, no vegetation, and the urinalysis was innocent. A thoracic CT scan (Figure 4) revealed bilateral pulmonary cavitations at the superior lobes, but bronchoalveolar lavage analysis was negative for bacteria, mycobacteria, fungi, and neoplastic cells.

Blood, stool, and eye exudate cultures were all negative.

**Figure 5:**
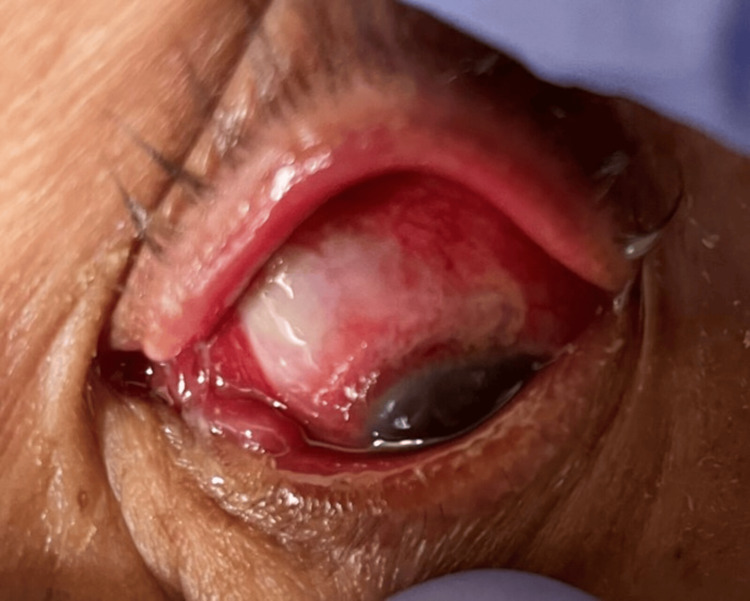
Spontaneous scleral perforation on a suppurated abscessed lesion

On day seven, a spontaneous scleral perforation occurred on a suppurated abscessed lesion (Figure 5), leading to progressive improvement in ocular motility, proptosis, and periorbital oedema.

**Figure 6:**
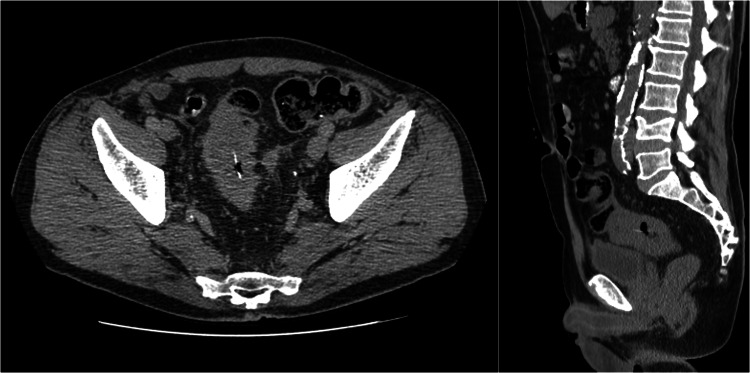
Abdomen and pelvic CT scan revealed a linear calcic foreign body at the distal sigmoid, with apparent wall thickening and a possible perforation.

Corticosteroid weaning was started. On day 15, the patient developed mucoid diarrhoea, with *Clostridium difficile* testing negative. An abdomen and pelvis CT scan revealed an image suggestive of a linear calcic foreign body at the distal sigmoid, with apparent wall thickening, probably of an inflammatory nature, and a possible perforation (Figure 6).

Imaging also revealed nodular cystic prostatic lesions, with transrectal ultrasound revealing benign prostatic hyperplasia with nodular prostatitis (normal prostate-specific antigen (PSA) levels).

**Figure 7:**
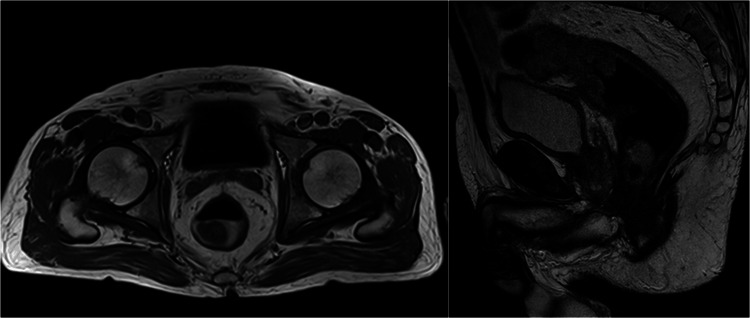
Magnetic resonance imaging revealed an 8 cm lesion at the rectosigmoid junction, with stenosis, perforation, and a vesical hypersignal.

The general surgery department decided to start IV metronidazole (800 mg three times a day). Magnetic resonance imaging revealed an 8 cm lesion at the rectosigmoid junction, with stenosis, perforation, and a vesical hypersignal (Figure 7).

**Figure 8:**
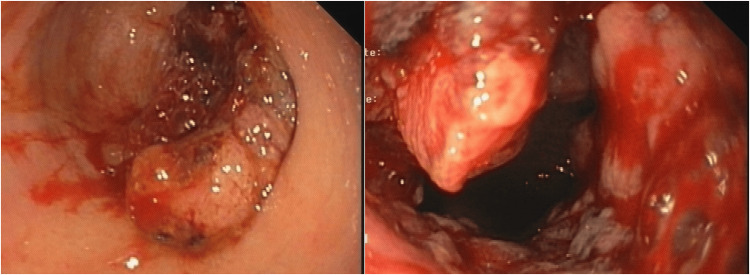
Colonoscopy revealed a neoplastic lesion with proximal stenosis

A colonoscopy identified a neoplastic lesion with proximal stenosis (Figure 8), and a biopsy confirmed the presence of an adenocarcinoma of the rectum.

The patient underwent anterior rectal resection with partial mesorectal excision, partial cystectomy and sigmoidectomy with end colostomy. Histopathology confirmed a pT4b B0 R0 rectal adenocarcinoma with a rectovesical fistula.

Following surgery, the patient was discharged in stable condition two weeks later. Four months of chemotherapy ensued, and he is now awaiting reconstructive colon surgery. The left eye progressed to phthisis bulbi, with no light perception or pain.

## Discussion

Panophthalmitis is a severe infection involving all ocular structures along with surrounding orbital and periorbital tissues [4]. An endogenous aetiology, though rare (affecting 0.5% of patients with fungemia and 0.04% with bacteremia [10]), is associated with a poor prognosis, often worse than exogenous causes [11]. Patients typically present with symptoms such as significant eyelid oedema, proptosis, hypopyon, restricted ocular movements, and elevated intraocular pressure. In our case, the patient exhibited most of these features and presented with no light perception, indicating a severe visual prognosis from the outset.

While a substantial proportion of patients with endogenous panophthalmitis require evisceration to control the infection and alleviate pain [12], our patient experienced spontaneous scleral rupture due to necrotizing scleritis, a rare complication of endophthalmitis [13]. This rupture led to a reduction in intraocular pressure and improvement in inflammatory symptoms, ultimately avoiding the need for surgical intervention.

This case underscores the importance of meticulous investigation when panophthalmitis is observed without any clear exogenous risk factors. Such cases often hold implications for both ocular and systemic prognosis. The absence of systemic symptoms should not deter a thorough evaluation, as extraocular infection foci, including endocarditis, gastrointestinal infections (e.g., liver abscesses), urinary tract infections, and indwelling catheters, are commonly reported sources [12]. Although rare, endogenous panophthalmitis may serve as a harbinger of occult colorectal cancer, even in the absence of a fistula, due to hematogenous spread through mucosal defects in the tumour [14, 15].

Microbiological investigations, such as staining and culturing ocular specimens, are essential for identifying the causative pathogen, though they should not delay the initiation of empirical antibiotic therapy. While current management guidelines recommend a "tap and inject" approach, the culture positivity rate for endogenous endophthalmitis remains low-approximately 28.6% overall and 0% after systemic antibiotic administration [16, 17]. In our case, the extremely elevated intraocular pressure and the impossibility of performing a vitreous tap at the emergency room precluded vitreous sampling, necessitating immediate initiation of systemic empirical antibiotic therapy. Blood cultures were also negative, consistent with literature showing a broad range of positivity rates (0%-100%), often influenced by prior antibiotic use [18].

No standardized treatment guidelines exist for endogenous endophthalmitis. Intravenous antibiotics are mandatory in all cases due to the hematogenous nature of the infection, distinguishing it from exogenous endophthalmitis [18]. Empirical therapy typically includes a combination of antibiotics, as administered in our patient, targeting both gram-positive and gram-negative organisms. Intravitreal antibiotic injections (e.g., vancomycin 1 mg/0.1 mL and ceftazidime 2.25 mg/0.1 mL) provide localized treatment and have been associated with better outcomes when administered early (within 24 hours) [19]. However, in our case, the poor visual prognosis and elevated intraocular pressure precluded local treatment.

The role of corticosteroids in endophthalmitis remains controversial. Their use in our case aimed to mitigate severe orbital inflammation. Uncontrolled inflammation can exacerbate retinal damage, and corticosteroids offer anti-inflammatory benefits. However, evidence of their efficacy is limited, and judicious use is advised [19].

## Conclusions

Endogenous panophthalmitis is a rare and severe condition that necessitates a high degree of clinical suspicion for timely diagnosis and management. This case underscores the critical importance of thorough investigative workup, as identifying the underlying cause can be pivotal not only in managing the ocular condition but also in safeguarding the patient’s life. Although vision could not be preserved in this instance, the comprehensive evaluation led to the early detection of occult colorectal cancer, prior to the development of local or distant metastases, significantly improving the patient’s prognosis and survival.
